# Impact of Health Education Sessions on Mothers' Quality of Life in Postpartum Period: Results of a Multicenter Randomized Controlled Trial

**DOI:** 10.1002/hsr2.71372

**Published:** 2025-10-22

**Authors:** Nazli Alafchi, Mojtaba Norouzi, Mostafa Eghbalian

**Affiliations:** ^1^ Hamadan University of Medicine and Health Sciences Hamadan Iran; ^2^ Department of Biostatistics and Epidemiology, School of Public Health Kerman University of Medical Sciences Kerman Iran; ^3^ Department of Epidemiology and Biostatistics, Faculty of Public Health, Social Determinants of Health Research Center Gonabad University of Medical Sciences Gonabad Iran

**Keywords:** counseling, female, postpartum mothers, quality of life, sexual behavior

## Abstract

**Background and Purpose:**

The postpartum period involves substantial physical, emotional, and social changes that can adversely affect the quality of life (QoL) and overall health status of mothers. This study aimed to assess the effect of health education sessions on mothers' QoL in the postpartum period.

**Methods:**

We conducted a clinical trial involving 112 first‐time mothers who had undergone vaginal delivery. Participants were recruited from healthcare centers in Hamadan, Iran. We selected case and control groups, using the cluster sampling method in two stages. We used two questionnaires: one to collect socio‐demographic data (e.g., mother's age, employment, education, housing, pregnancy planning, newborn's gender, abortion history), and another (Mother‐Generated Index) to assess postpartum QoL through 30 questions across 8 childbirth‐related dimensions, rated on a 5‐point Likert scale with total scores from 0 to 20. The health education for health promotion was provided to the interventional group throughout three sessions, held weekly. The aspects of the QoL of both groups were compared before and after health education.

**Findings:**

The findings revealed a notable association in the overall QoL scores of mothers, with the case group showing a significant improvement post‐health education sessions, compared to the control group (*p* < 0.001). Only the case group exhibited significant changes in self‐perception scores pre‐ and post‐intervention (*p* < 0.001). Additionally, within the case group, there was a significant improvement in positive attitudes and comfort regarding postpartum sexual relations after the intervention compared to the control group (*p* = 0.007). Nonetheless, no notable correlations were found in other dimensions of QoL.

**Conclusion:**

Health education sessions have the potential to improve the QoL scores of mothers in the postpartum period. Interventions focusing on the mother's self‐perception and feelings towards sexual relations have a greater impact on QoL. Therefore, counselors should prioritize these aspects during this period.

## 1 Introduction

While prenatal care is often prioritized, the postpartum period frequently lacks sufficient attention [1]. This period, known as the fourth trimester of pregnancy, marks a significant transition in women's lives, encompassing physical changes as they embrace the responsibilities of motherhood. Additionally, psychological and familial change occur as the newborn becomes integrated into family life [2]. The postpartum period is recognized as a critical phase for the well‐being of the mother, baby, and family [3]. While postpartum care schedules vary, many countries adhere to standard care packages lasting 6 weeks post‐delivery [4]. Despite this, postpartum care often concludes while mothers are still adjusting to their new roles [5]. While some women navigate the physiological, psychological, and social changes following pregnancy and childbirth with ease, others may encounter difficult challenges [6].

Regular treatment and routine care for postpartum mothers in Iran involve comprehensive assessments and support during the critical first 6 weeks after childbirth to improve maternal and newborn health outcomes [7]. Postpartum care traditionally receives less attention compared to prenatal care, resulting in inadequate coverage and variable quality, with an average coverage of about 74% in Iran [8]. The care includes evaluating physical and mental health issues of the mother, health education sessions on breastfeeding, infant care, family planning, nutrition, and activity, as well as prevention of postpartum depression [9].

Managing the changes experienced during the postpartum period significantly influences the quality of life (QoL) and health status of mothers [10]. QoL is a crucial measure for evaluating health services [11] and is influenced by various factors such as physical, psychological, relational, and social well‐being [12]. The QoL experienced by postpartum mothers, their infants, and families plays a crucial role in enhancing maternal functioning and the care provided to infants [13]. Enhancing postpartum QoL has a beneficial impact on the health relationships within the family as well as on the developmental progress of the infant [14, 15]. Conversely, negative factors such as postpartum depression and anxiety can greatly diminish a mother's QoL, leading to disrupted bonding with the infant, poorer infant nutrition, and challenges in overall family well‐being [16].

Various factors contribute to a decrease in postpartum QoL, with sociocultural influences playing a significant role [17]. For example, regarding sexual relations, postpartum mothers in Iran often face challenges such as decreased sexual desire, vaginal dryness, and discomfort, which can impact their intimate relationships and overall QoL. These sexual health issues are compounded by cultural and social factors, underscoring the need for sensitive healthcare interventions that address both physical recovery and sexual well‐being to foster better postpartum outcomes for mothers and their families [14, 15]. Ensuring comprehensive postpartum care that includes attention to sexual health is vital for improving life quality and family dynamics in the postpartum period in Iran.

Health education sessions are recognized as a valuable intervention for empowering individuals to enhance their QoL [18]. Consultation serves as a process designed to aid individuals in navigating crises and developing effective coping strategies. Through health education, active listening, mutual understanding, responsive communication, and targeted interventions can be facilitated, offering potential benefits to mothers. While women may be able to resume many of their pre‐birth roles upon returning to their functional status, the postpartum period often involves significant changes that can make a full return challenging for many [19]. As such, there is a clear need for interventions like health education sessions, which have proven to be a beneficial and enduring approach that can enhance individual awareness and QoL.

In Iran, predictors influencing postpartum QoL are not well investigated, with insufficient studies conducted on this topic [1]. Furthermore, due to the cultural and environmental differences that can impact the QoL, findings from studies conducted in other countries cannot be readily generalized to Iran. Although health education sessions have been shown to benefit postpartum mental health in Iran, there remains a shortage of multicenter randomized controlled trials that assess their impact on the overall QoL of postpartum mothers. Therefore, this study aims to evaluate how health education sessions affect the QoL of mothers during the postpartum period.

## 2 Materials and Methods

### 2.1 Study Design and Setting

We conducted a parallel‐group, multicenter randomized controlled trial involving 112 primiparous mothers. The study was conducted at several health centers affiliated with Hamadan University of Medical Sciences, located in Hamadan, Iran. These centers provide routine postpartum care services to a diverse population of mothers from both urban and suburban areas.

### 2.2 Ethical Considerations

The study protocol was approved by the Hamadan ethical committee, under approval number (ID: 9408124396). Written informed consent was obtained from all participants before their inclusion in the study. Participation was voluntary, and confidentiality of participants' information was maintained throughout the research process. The study was registered at the Clinical Trial Center of Iran (IRCT2016030510426N10) and conducted under the ethical standards outlined in the Declaration of Helsinki.

### 2.3 Sampling

The sampling approach utilized a two‐stage cluster random sampling method. Hamadan City was divided into five distinct regions based on geographical directions: north, south, east, west, and central. In total, Hamadan City has 20 health centers distributed across the five regions (with approximately four centers per region). From these, we randomly selected two health centers in each region, resulting in a total of 10 health centers included in the sample. Of these 10 centers, five were randomly assigned to the case group and five to the control group. The health education services at these health centers were provided by trained health professionals, including midwives, who are qualified to deliver the specific interventions required for the study.

### 2.4 Inclusion and Exclusion Criteria

The inclusion criteria for this study included primiparous mothers who could read and write in Persian, and their healthy newborns weighing between 2500 and 4000 g. “Healthy newborns” were defined as term infants without significant medical comorbidities or complications appropriate for their gestational age. Additional inclusion criteria were the absence of physical and mental illnesses in the mothers, no obstetric complications during the current pregnancy, no participation in other educational programs, and delivery by vaginal birth. Mothers and infants who were hospitalized for more than 48 h following delivery, as well as mothers who missed two or more health education sessions, were excluded from the study. Participants from both groups completed questionnaires through interviews, and mothers who had undergone cesarean sections were excluded from the study, with replacements following the specified method. There were no modifications made to the methods post‐trial initiation.

### 2.5 Randomization

Participants were assigned to either a case group or a control group at random. The control group received standard care, while the case group received standard care in addition to health education sessions. The selection was conducted using a random sampling method. The allocation process was carried out using a permuted‐block randomization design by a team member not involved in the selection process. Each participant was assigned a code in sealed envelopes to maintain confidentiality during the assignment process. The participants were divided into Groups A and B based on a predetermined sequence, with the allocation ratio being 1:1. The data analyst was blinded to the interventions provided to each group. There were 10 small groups in the experimental group, each consisting of 5–10 individuals.

### 2.6 Data Collection Tool

We used two questionnaires for data collection. The first questionnaire gathered socio‐demographic information about the participants, including details such as the mother's age, employment status, education level, housing situation, pregnancy planning, newborn's gender, and abortion history. The second questionnaire focused on postpartum QoL. Previous studies have confirmed the validity and reliability of this tool for the Iranian context [20, 21]. It consists of 30 questions across 8 dimensions related to childbirth and child‐rearing, covering aspects such as the mother's self‐perception, feelings towards her child and others, sexual relations, physical health, and satisfaction with the delivery method. A 5‐point Likert scale was utilized to evaluate each question, with scores ranging from a minimum of 0 to a maximum of 4. Additionally, the highest QoL score was recorded as 20, indicating an improved QoL, while the lowest score remained at 0. The questionnaire's content validity was assessed by experts and deemed acceptable (Cronbach's alpha = 0.84).

### 2.7 Health Education Interventions

The experimental group participated in health education sessions conducted in small groups (5–10 individuals) at the health center, lasting between 3 and 60 min. These sessions were held at specific intervals following childbirth: the first session occurred 3–5 days postpartum, covering topics such as physiological changes, signs of danger, sexual health, lactation, and nutrition. The second session took place 10–15 days postpartum, focusing on stress recognition, coping strategies, relaxation techniques, and factors affecting relationships. The third session was held 17–20 days postpartum, addressing physical activity, appropriate exercises, self‐actualization, spiritual well‐being, and the benefits of maintaining good health. The researcher provided weekly follow‐ups for 3 weeks after the health education sessions and shared contact information for any queries. The health education sessions were delivered by trained health professionals, including midwives, who received specific training on the intervention protocol conducted by our research team before the study. To ensure adherence and fidelity, regular supervision and monitoring were implemented throughout the intervention period, including periodic observations and checklist assessments of the sessions. Participants also received a booklet with advice from scientific sources. Mothers from both groups completed questionnaires during routine postnatal care visits 42 days after childbirth.

In this study setting, the usual postpartum care for mothers typically includes routine clinical assessments and health education during postnatal visits at the health center. These visits focus on physical examination of the mother and newborn, assessment of vital signs, monitoring breastfeeding status, immunization updates, and general health advice. Education topics commonly covered are basic newborn care, breastfeeding support, nutrition, hygiene, and recognition of postpartum warning signs. Unlike the experimental group, these standard care visits are generally individual rather than conducted in small group health education sessions. No structured health education or follow‐up calls were routinely provided beyond the scheduled visits. The control group received the educational booklet only after completion of the study to ensure equitable access to the information.

### 2.8 Sample Size

Based on a prior study [22] given that factored in a Type I error rate of 5%, a Type II Error (β) rate of 20%, and an attrition rate of 10%, the required sample size for each case and control group was determined to be 60.

### 2.9 Statistical Analysis

The Kolmogorov‐Smirnov test confirmed that all quantitative variables were normally distributed. To examine the heterogeneity of demographic variables within the group, Amon's Chi‐score or Fisher's exact test was used. To assess the differences between groups, an independent *t*‐test was used. For intra‐group comparisons before and after the intervention, a paired *t*‐test was used, or in the case of inhomogeneity, an analysis of covariance was used. To control for confounding factors, an analysis of covariance was used. The significance level in all tests was 95%.

The QoL variables were measured using multiple Likert‐scale items, each with five response options. These individual items were combined to generate composite scores representing different dimensions of QoL. Although Likert‐scale data are ordinal, the composite scores approximate continuous interval‐level data, allowing for the use of parametric statistical tests. Therefore, the paired *t*‐test was employed to compare pre‐ and post‐intervention composite scores within groups. Although the control group did not receive the intervention, we conducted pre‐ and post‐assessments in this group to monitor any changes that might occur over time due to natural variation or external influences. This approach allows us to confirm that any significant changes observed in the case group are attributable to the intervention rather than other factors. All statistical analyses were performed using SPSS software version 20.

## 3 Results

**Figure 1:**
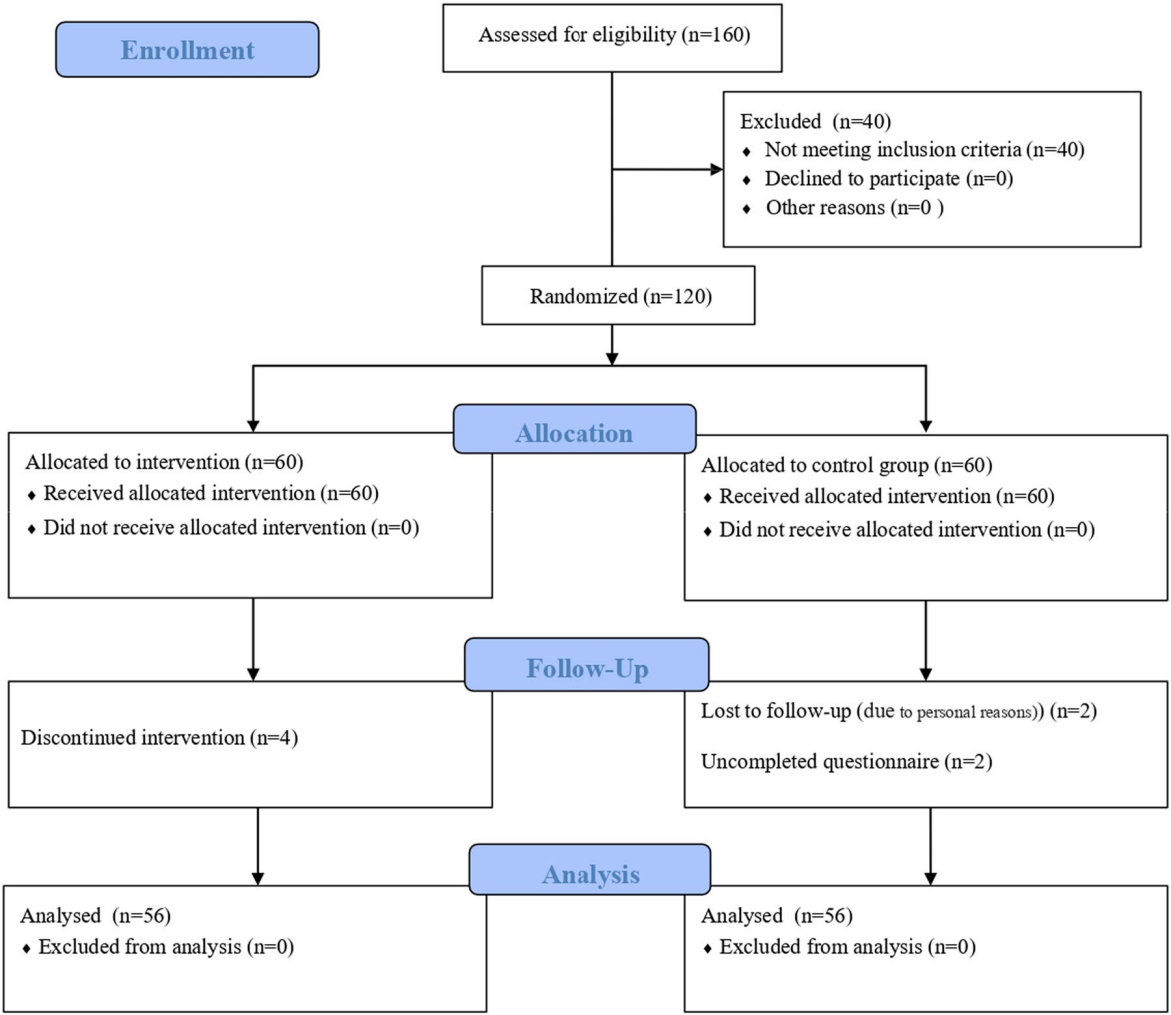
Consort flow diagram.

We enrolled 160 women who completed informed consent forms to assess their eligibility. Out of these, 40 women were deemed ineligible based on the criteria and were excluded. Consequently, 60 women were assigned to each group. After the follow‐up period, 56 postpartum women from the experimental group and 56 from the control group were included in the analysis (Figure 1).

The average age of mothers in the intervention group was 23.46 ± 4.75, while in the control group, it was 23.77 ± 4.98. Fisher's exact test was employed to assess the equality of variables in job and education between the experimental and control groups, while for other variables, the Chi‐square test was applied. The case and control groups showed homogeneity across all relevant variables (Table 1).

**Table 1:** Features of mother's socio‐demographic in the case and control groups.

Variables	Frequency (%)		*p* value
	Case (*N* = 56)	Control (*N* = 56)	
Mother's education			
High school	22 (39.3)	23 (41.1)	0.965
Diploma	18 (32.1)	17 (30.4)	
Associate degree	6 (10.7)	6 (10.7)	
Bachelor	10 (17.9)	10 (17.9)	
Abortion experience			
Yes	8 (14.3)	6 (10.7)	0.775
No	48 (85.7)	50 (89.3)	
Planned pregnancy			
Yes	46 (82.1)	43 (76.8)	0.641
No	10 (17.9)	13 (32.2)	
Newborn gender			
Male	35 (62.5)	31 (55.4)	0.565
Female	21 (37.5)	25 (44.6)	
Vaginal delivery			
With episiotomy	36 (64.3)	39 (69.6)	0.821
Without episiotomy	10 (17.9)	9 (16.1)	
With tear	10 (17.9)	8 (14.3)	
			
Age (years)	Mean ± SD		0.742
	23.46 ± 4.75	23.77 ± 4.98	

*Note:* Chi‐square test/Independent *t*‐test.

Table 2 shows the examination of the difference in the average factors of the QoL of mothers in the postpartum period before and after the intervention. The results of Table 2 are listed below.

**Table 2:** Difference in the of the quality‐of‐life factors of mothers in the postpartum period before and after the intervention.

Variables	Before	After	*p* value^a^
Mother's feelings about the child			
Case	10.66 ± 1.62	10.88 ± 1.49	0.805
Control	9.96 ± 1.81	10.20 ± 1.84	
*p* value^b^	0.025	0.030	
The effect of childbirth on the economic status of the family			
Case	3.45 ± 1.04	3.22 ± 1.04	0.526
Control	2.96 ± 1.13	3.23 ± 1.17	
*p* value^b^	0.003	0.914	
Satisfaction with the delivery method			
Case	2.96 ± 1.17	3.02 ± 1.15	0.210
Control	2.45 ± 1.33	2.46 ± 1.31	
*p* value^b^	0.017	0.009	
Choosing the delivery method in the case of rebirth			
Case	2.54 ± 1.35	2.73 ± 1.24	0.068
Control	3.36 ± 1.07	3.59 ± 1.08	
*p* value^b^	0.001	0.001	
	**Before**	**After**	***p* value** c
The mother's feeling about herself			
Case	16.04 ± 2.75	18.43 ± 2.54	< 0.001
Control	15.91 ± 2.57	15.66 ± 2.73	0.137
*p* value^b^	0.804	< 0.001	
The mother's feeling about her husband and others			
Case	22.34 ± 3.98	23.73 ± 3.48	0.128
Control	21.87 ± 3.65	22.25 ± 3.83	0.070
*p* value^b^	0.521	0.488	
The mother's feeling towards sexual relations			
Case	4.85 ± 2.95	7.75 ± 2.80	< 0.001
Control	5.16 ± 3.00	6.25 ± 3.01	0.015
*p* value^b^	0.591	0.007	
Physical health status			
Case	17.70 ± 5.22	20.78 ± 4.70	< 0.001
Control	18.59 ± 5.66	19.73 ± 4.89	0.044
*p* value^b^	0.387	0.351	
Quality of life (total)			
Case	80.53 ± 11.63	89.55 ± 10.28	< 0.001
Control	80.26 ± 10.81	83.37 ± 9.41	< 0.001
*p* value^b^	0.790	0.001	

^a^ Paired *t*‐test.

^b^ Independent *t*‐test.

^c^ Analysis of covariance.

**Mother's feelings about the child:** The difference between the mean of the case and the control before and after the intervention is significant (*p* < 0.05). Based on the results of the analysis of covariance, the effect of the group after the intervention, with the adjustment of the effect before the intervention, is not significant (*p* = 0.805).

**The effect of childbirth on the economic status of the family:** The analysis of covariance indicates that the difference in averages between the case and control groups before and after the intervention is significant (*p* < 0.05). However, when adjusting for the effects before the intervention, the impact of the group after the intervention is not significant (*p* = 0.526).

**Satisfaction with the delivery method:** The difference between the case and control averages before and after the intervention is significant (*p* < 0.05). According to the results of the analysis of covariance, the effect of the group after the intervention, with the adjustment of the effect before the intervention, is not significant (*p* = 0.210).

**Choosing the delivery method in the case of rebirth:** The difference between the case and control averages before and after the intervention is significant (*p* < 0.05). According to the results of the analysis of covariance, the effect of the group after the intervention, with the adjustment of the effect before the intervention, is not significant (*p* = 0.068).

**Mother's feelings about herself:** The difference between the average of the case and the control before the intervention was not significant (*p* = 0.804), according to the results of the paired *t*‐test, in the case group, the average increased from 16.04 to 18.43 (*p* < 0.001) and in the control group, the difference between the mean before and after the intervention is not significant (*p* = 0.137).

**Mother's feeling about her husband and others:** The difference between the average of the case and the control before the intervention was not significant (*p* = 0.521), according to the results of the paired *t*‐test, in the case group, the average increased from 22.34 to 23.73 (*p* = 0.128) and in the control group, the difference between the mean before and after the intervention is not significant (*p* = 0.070).

**Mother's feeling towards sexual relations**: The difference between the average of the case and the control before the intervention was not significant (*p* = 0.591), according to the results of the paired *t*‐test, in the case group, the average increased from 4.85 to 7.75 (*p* < 0.001) and in the control group, the average difference increased from 5.16 to 6.25 (*p* = 0.015).

**Physical health status:** The difference between the case and control average before the intervention was not significant (*p* = 0.387), according to the results of the paired *t*‐test. In the case group, the average increased from 17.70 to 20.78 (*p* < 0.001, effect size = 0.62), and in the control group, the difference between the mean before and after the intervention is also significant; the average increased from 18.59 to 19.73 (*p* = 0.044, effect size = 0.21).

**QoL (total):** The difference between the case and control average before the intervention was not significant (*p* = 0.790), according to the results of the paired *t*‐test, in the case group, the average increased from 80.53 to 89.55 (*p* < 0.001, effect size = 0.82) and in the control group, the average difference increased from 80.26 to 83.37 (*p* < 0.001, effect size = 0.31).

### 3.1 Reporting of Adverse Events and Safety Monitoring

Throughout the study period, participants were closely monitored for any adverse events or unintended effects associated with the health education intervention. No adverse events were reported by participants or identified by the research team. Given the low‐risk nature of the intervention, this finding aligns with expectations. Nonetheless, the absence of adverse effects highlights the safety and tolerability of the health education program.

## 4 Discussion

The study evaluated the effects of health education on the QoL of postpartum mothers. Results indicated that the test group, which received health education, experienced a significant improvement in QoL scores compared to the control group. Notably, self‐perception scores improved in the test group after the intervention, and there was also an increase in positive attitudes toward sexual relations. However, while both groups showed an increase in physical health status scores, this change was not statistically significant.

Given that childbirth and the challenges associated with caring for a baby can impact the QoL of mothers, it is crucial to focus on their well‐being during the postpartum period [12]. This study aligns with previous research findings and highlights the significant impact of interventions on maternal QoL [23, 24, 25]. After the intervention, we observed a significant increase in the average QoL scores in the intervention group compared to the control group. However, these results diverged from the findings of Dodd et al. [26]. Health education improves QoL through multiple pathways. It provides emotional support and psychoeducation to reduce stress and depressive symptoms, enhancing mental well‐being. Health education also strengthens social support and improves communication, reducing isolation and fostering better family relationships. Additionally, it helps mothers reframe negative thoughts and build coping skills, promoting positive self‐perception and overall health. Differences in intervention timing, delivery, and measurement explain variations in study findings.

The study findings regarding mothers' self‐perception revealed a significant difference in mean scores within the intervention group, aligning with similar results found in other studies [22, 27]. In a study by Sadat et al. [20], it was found that there is no significant difference in mothers' self‐perception between those who had a caesarean section and those who had a vaginal delivery when health education is not provided [20]. Thus, by implementing health education and improving a mother's self‐perception, there is potential to enhance the QoL for mothers during the postpartum period.

There was a significant increase in the mean scores in the field of mothers' feelings toward sexual relations after the intervention in the test group compared to the control group. These results were consistent with the findings of Karimi et al. [28]. Changes in anatomy, hormonal balance, family dynamics, and marital relationships following childbirth impact postpartum sexual function [29]. Women's sexual health is a vital part of life at any age. There is evidence that sexual function decreases during pregnancy and does not return to its original level during the postpartum period [30]. Health education enhances postpartum sexual relations by addressing psychological, relational, and physiological factors. It helps reduce anxiety and stress related to body changes and hormonal shifts, improving sexual desire, arousal, and satisfaction. Health education also builds sexual self‐efficacy by correcting misconceptions and teaching coping strategies, fostering positive attitudes toward intimacy. Additionally, it improves communication and emotional connection with partners, which strengthens marital relationships and supports healthier sexual function after childbirth.

While the disparity on average scores related to physical health status between the test and control groups was not statistically significant, both groups exhibited an increase on average scores post‐intervention. These findings diverged from those reported by Garcia et al. [31]. The rise in scores in both groups following the intervention can be attributed to the gradual physical recovery process and the restoration of physical well‐being to pre‐pregnancy levels. Nonetheless, the intervention group showed a greater increase on average scores, likely due to the impact of health education.

In no other aspect of the QoL questionnaire, a significant association found between the intervention and control groups. For instance, in terms of how mothers feel towards their husbands and others, similar to previous studies [20, 22, 32] no significant association was observed. This may indicate that health education was not effective for certain reasons, such as a limited number of health education sessions or insufficient guidance on this particular aspect.

### 4.1 Study Limitations

This study has several limitations. First, the intervention program could not be sustained over an extended period due to the postpartum condition of the mothers, whereas behavior change programs typically require ongoing training and reinforcement over time. Second, the study was conducted exclusively among an urban population, which may limit the generalizability of the findings. Additionally, while the intervention group received structured health education sessions, the control group received only standard postpartum care without additional contact. It is well established that increased attention or contact during the postpartum period can itself improve outcomes. Therefore, the absence of an attention‐matched control group may limit our ability to fully isolate the effects of the health education intervention from those of additional contact. Future studies should consider including an attention‐control group to better differentiate between the impact of health education content and the influence of participant contact. Another limitation of this study is the restrictive inclusion and exclusion criteria, which limited participation to healthy primiparous mothers and excluded those with pregnancy complications or other significant health issues. While this approach was necessary to ensure internal validity and accurately assess the efficacy of the intervention in a controlled setting, it limits the generalizability of the findings to the broader postpartum population. Mothers who might benefit most from such counseling, including high‐risk or medically complex cases, were not represented in this study. Future research should aim to evaluate the effectiveness of similar interventions in more diverse and high‐risk groups to better understand their impact across different populations.

A further limitation of this study is the slightly smaller final sample size than planned, with 56 participants per group analyzed instead of 60. This reduction may have modestly reduced statistical power, which should be considered when interpreting the results.

### 4.2 Strengths of the Study

This multicenter randomized controlled trial benefits from several notable strengths. First, the relatively large sample size of 112 first‐time mothers enhances the statistical power and reliability of our findings. Second, the use of a cluster sampling method and recruitment from multiple healthcare centers in Hamadan increases the generalizability of results within similar populations. Third, the health education intervention was structured into 3 weekly sessions, providing sustained support during the critical postpartum period. Additionally, we employed validated, multidimensional questionnaires to comprehensively assess QoL, including self‐perception and sexual relation domains, which allowed for nuanced evaluation of health education effects. These strengths collectively contribute to the robustness and relevance of our findings on improving maternal QoL through health education.

## 5 Conclusion

Providing health education to mothers during the postpartum period has been shown to enhance overall QoL scores. To enhance the QoL for women in the postpartum period, healthcare providers can focus on health education interventions that address the mother's self‐perception and sexual well‐being. Such interventions not only benefit women during the postpartum period but also contribute to the health and QoL of families, including partners and infants. It is recommended for future research to explore alternative health education approaches, as well as replicate this study in rural communities and among diverse ethnic groups.

## Author Contributions

Study concept and design: Nazli Alafchi and Mostafa Eghbalian. Acquisition of data: Nazli Alafchi and Mostafa Eghbalian. Analysis and interpretation of data: Nazli Alafchi and Mostafa Eghbalian. Drafting of the manuscript: Nazli Alafchi, Mostafa Eghbalian, and Mojtaba Norouzi. Critical revision of the manuscript for important intellectual content: Nazli Alafchi, Mostafa Eghbalian, and Mojtaba Norouzi. Statistical analysis: Mostafa Eghbalian. Administrative, technical, and material support: Nazli Alafchi, Mostafa Eghbalian, and Mojtaba Norouzi. Study supervision: Nazli Alafchi.

## Conflicts of Interest

The authors declare no conflicts of interest.

## Transparency Statement

The lead author Mostafa Eghbalian affirms that this manuscript is an honest, accurate, and transparent account of the study being reported; that no important aspects of the study have been omitted; and that any discrepancies from the study as planned (and, if relevant, registered) have been explained.

## Data Availability

The data that support the findings of this study are available from the corresponding author upon reasonable request.
